# Ultrasound and electrical nerve stimulation-guided S1 nerve root block

**DOI:** 10.1007/s00540-013-1591-y

**Published:** 2013-03-14

**Authors:** Masaki Sato, Yasuhito Mikawa, Akiko Matuda

**Affiliations:** Department of Anesthesiology, Atago Hospital, Atago 1-4-13, Kouchi, Kouchi 780-0051 Japan

**Keywords:** Ultrasound, Nerve stimulation, Nerve root block

## Abstract

A selective lumbosacral nerve root block is generally is performed under X-ray fluoroscopy, which has the disadvantage of radiation exposure and the need for fluoroscopy equipment. In this study, we assessed the effectiveness of ultrasound and nerve stimulation-guided S1 nerve root block on 37 patients with S1 radicular syndrome. With the patient in a prone position, an ultrasound scan was performed by placing the probe parallel to the body axis. The needle was pointed slightly medial from the lateral side of the probe and advanced toward a hyperechoic area in the sacral foramina with ultrasound guidance. Contrast medium was then injected and its dispersion confirmed by fluoroscopy. The acquired contrast images were classified into intraneural, perineural, and paraneural patterns. The significance of differences in the effect of the block among the contrast image patterns was analyzed. After nerve block, decreased sensation at the S1 innervated region and pain relief was achieved in all patients. No significant difference was noted in the effect of the block between perineural and paraneural patterns. In conclusion, this technique provided reliable S1 nerve root block in patients with S1 radicular syndrome and minimized radiation exposure.

A selective lumbosacral nerve root block is a useful peripheral nerve block that is frequently applied to diagnose and treat pain associated to the nerve root. It is generally performed under X-ray fluoroscopy [1–3], which has two major disadvantages: exposure of the patient to radiation exposure and the need for fluoroscopy equipment.

We recently reported that an L5 nerve root block can be performed safely and reliably under ultrasound guidance and electrical nerve stimulation [4]. In the study reported here, we evaluated the effectiveness of S1 nerve root block in the same way.

The study protocol was approved by the Institutional Review Board of Atago Hospital, and informed consent was obtained from all study participants. Thirty-seven patients (29 males, 8 females; mean age 46.5 ± 17.0 years, age range 20–78 years) with S1 radiculopathy were included in this study. S1 radiculopathy was defined as disease characterized by weakness in the flexor hallucis longus and/or gastrocnemius, hypo- or areflexia at the Achilles tendon and hypoflexia or anesthesia along the S1 dermatome, as well as compression of the S1 nerve as evidenced on magnetic resonance imaging or computed tomography scans. Twenty-six patients were diagnosed with lumbar disc herniation and 11 with lumbar spinal canal stenosis. Patients who had previously undergone lumbar spinal surgery and those with lumbosacral malformation were excluded. The patient was placed in a prone position with a pillow under the lower abdomen to orient the sacrum in a horizontal position. Following aseptic preparation of the puncture site, a curved ultrasound probe (C60e 5-2 MHz; Sonosite Micromax, Sonosite, Bothell, WA) was placed in its sterile plastic bag with ultrasound gel, and the probe was positioned longitudinally to the parasacral area, approximately 2 cm lateral to the midline to identify the articular processes (AP) of the lower lumbar vertebrae and posterior sacral surface. The AP observed at the extreme caudal side corresponds to the L5/S level, and the concavity at the posterior sacral surface located at a slightly caudal site is the S1 posterior sacral foraminen. The probe was inclined mediocaudally along the slope of the S1 sacral foraminen, via which a hyperechoic area is observed in most cases (Fig. 1). This structure was assumed to be the S1 nerve root and was targeted in this technique. When this structure could not be observed, the posterior sacral foramina (SF) were targeted. We used the out-of-plane approach. An insulated 70- or 100- mm 21G nerve stimulating needle (Type CCR; Hakko, Tokyo, Japan) was pointed slightly medial from the lateral side of the probe and advanced toward the target with ultrasound guidance (Fig. 2). The entire view of the needle could not be confirmed by ultrasonography. However, the depth of the needle tip could be assumed from the degree of tissue deformation around the tip even though the needle tip was not present in the beam. When the tilt of the probe or needle was adjusted in this state, the needle tip could be visualized. When the needle tip struck the posterior sacral surface, we withdrew the needle slightly and redirected it more mediolaterally. When the needle passed through the posterior SF, electrical stimulation was applied to elicit motor and sensory response with a nerve stimulator (Stimuplex; B Braun, Bethlehem, PA) set at 1 mA. The needle was advanced slowly so as not to strike the nerve root and halted when the patients reported a tapping sensation in the gluteal region or lower limbs corresponding to the frequency of the electrical stimulation. At this time, 0.5 ml of 1 % lidocaine was slowly injected, followed by 1 ml of contrast medium, iohexol (omnipaque240R; Daiichi-Sankyo, Tokyo, Japan) injection. The distribution of these fluids was confirmed by fluoroscopy. The contrast-enhanced images thus acquired were classified into three patterns: (1) intraneural pattern, with homogeneous visualization of the contrast agent along the entire width of the nerve root; (2) perineural pattern, with contrast agent visualization around the nerve root; (3) paraneural pattern, with contrast agent visualization irrespective of the arrangement of the nerve root. Following the fluoroscopy, 2 ml of 1 % lidocaine and 2 mg dexamethasone sodium phosphate were injected, and the effect of the block was evaluated using a numerical pain score during walking (0 = no pain, 10 = the severity of pain before the block) after 1 h of rest. The intensity of the pain was categorized into three levels using a score of 0–10, with 0 = complete pain relief, 1–7 = alleviated pain, 8–10 = no pain relief. Additionally, the differences in the effect of the block among the contrast image patterns were analyzed using the Mann–Whitney *U* test. A value of *P* < 0.05 was considered to be statistically significant. Data were analyzed using the SPSS statistical package, ver. 10.0 (SPSS, Chicago, IL).

**Fig. 1:**
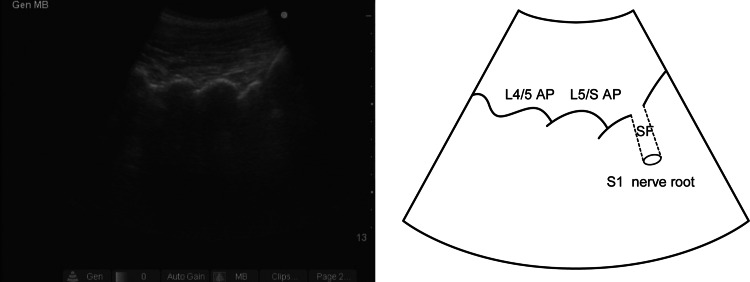
A long-axis view of the articular processes (AP), about 2-cm lateral to the median. A hyperechoic area in the sacral foramina (SF) is observed

**Fig. 2:**
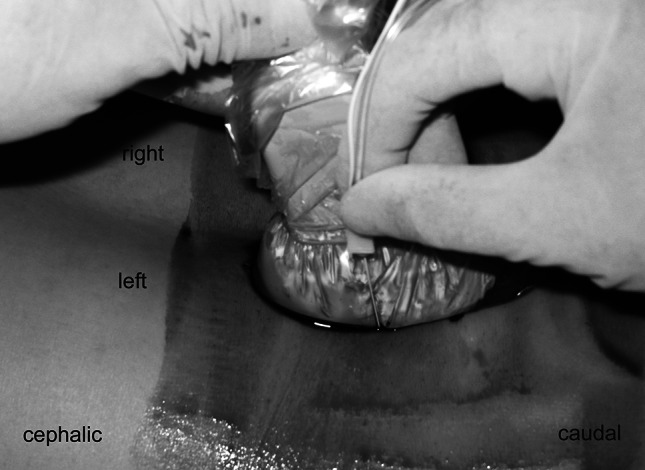
The position of patient, the needle, and the probe of the ultrasound apparatus under the left S1 nerve root block. The needle is pointed slightly medially from the lateral side of the probe and advanced toward the target with ultrasound guidance

There were no complications after the block. All patients reported a tapping sensation corresponding to electrical stimulation along the S1 dermatome. In addition, S1-innervated muscle (flexor hallucis longus, gastrocnemius, etc.) contraction was observed in three patients. The acquired contrast images showed intraneural, perineural, and paraneural patterns in zero, 33, and four patients respectively. Cases showing partial enhancement of the nerve sheath were included in the perineural pattern. While epidural enhancement was noted in all patients, intravascular enhancement was noted in two patients. The block was performed by correcting the needle tip position in these cases.

Hypesthesia and pain relief in the S1 innervated region was achieved in all patients. The relationship between the effect of the block and contrast image patterns is as follows. For the perineural pattern, complete pain relief was observed in 24 patients and alleviated pain was observed in nine patients. For the paraneural pattern, complete pain relief was observed in three patients and alleviated pain was observed in one patient. There was no evident difference in the effect of the block between the perineural and paraneural patterns.

Peripheral nerve blocks have been increasingly performed with ultrasound guidance in both operating rooms and outpatient clinics due to recent advancements in high-quality, portable ultrasound apparatus [5–9]. In general, an S1 nerve root block is performed under fluoroscopy by identifying the posterior SF. However, this identification is occasionally obscured due to the presence of intestinal gas. In such cases, ultrasonography allows the identification of the posterior SF independent of any effect of intestinal gas.

The hyperechoic area in the SF can be observed by aligning the probe axis with the SF inclination. Ultrasonography revealed the presence of a hyperechoic area in 32 patients (86 %). This hyperechoic area was assumed to be the S1 nerve root because a tapping sensation occurred consistently and instantaneously in patients when the needle tip was close to this area.

An important concern when using this procedure is the accurate identification of the vertebral level. Therefore, the location of the AP should be first confirmed on lateral-view lumbar vertebrae radiographs. Attention should also be paid to lumbosacral malformations, which may be present in some cases. In general, the L4–5 intertransverse level serves as the best indicator to identify the target level accurately. In some cases, a protruding posterior sacral surface may be misidentified as an AP.

An in-plane approach is generally employed to guide the needle for a peripheral nerve block because the position of the needle can be readily confirmed [10]. However, for an S1 nerve root block, the direction of needle advancement should be along the axis of the SF because the needle tip is advanced into the SF. For this reason, we employed an out-of-plane approach.

Radiating pain is not always essential in nerve root blocks under fluoroscopy. Often these blocks can lead to nerve injury, such as residual dysesthesia [11, 12]. Nerve root blocks can be performed more safely and reliably by using concomitant electrical nerve stimulation to guide needle advancement close to the nerve [13, 14]. This prevents the needle from directly striking the nerve root, regardless of whether the nerve root can be confirmed by ultrasonography.

No difference was detected in the effect of nerve blocks with the perineural or paraneural pattern, suggesting that the needle tip does not necessarily have to be close to the nerve root. However, the needle tip should be present on the ventral side of the ligament where the nerve root is located. Pfirrmann et al. [15] also classified the contrast images acquired during fluoroscopy-guided selective nerve root block and observed no significant difference at 15 min and 2 weeks after the block among the contrast image patterns.

In conclusion, this technique provided reliable S1 nerve root block in patients with S1 radicular syndrome and is useful in terms of avoiding radiation exposure.
